# Seismic and thermal precursors of crater collapses and overflows at Stromboli volcano

**DOI:** 10.1038/s41598-023-38205-7

**Published:** 2023-07-10

**Authors:** Flora Giudicepietro, Sonia Calvari, Walter De Cesare, Bellina Di Lieto, Federico Di Traglia, Antonietta M. Esposito, Massimo Orazi, Pierdomenico Romano, Anna Tramelli, Teresa Nolesini, Nicola Casagli, Pierfrancesco Calabria, Giovanni Macedonio

**Affiliations:** 1 Istituto Nazionale di Geofisica e Vulcanologia, Osservatorio Vesuviano, Via Diocleziano 328, 80124 Naples, Italy; 2 Istituto Nazionale di Geofisica e Vulcanologia, Osservatorio Etneo, Piazza Roma 2, 95125 Catania, Italy; 3 Università degli Studi di Firenze, Centro per la Protezione Civile, Piazza San Marco 4, 50121 Firenze, Italy; 4 Università degli Studi di Firenze, Dipartimento di Scienze della Terra, Via La Pira 4, 50121 Firenze, Italy; 5 National Institute of Oceanography and Applied Geophysics - OGS, Borgo Grotta Gigante 42/C, 34010 Sgonico (Trieste), Italy

**Keywords:** Volcanology, Natural hazards

## Abstract

Lava overflows are highly hazardous phenomena that can occur at Stromboli. They can destabilize the crater area and the “Sciara del Fuoco” unstable slope, formed by several sector collapses, which can generate potentially tsunamigenic landslides. In this study, we have identified precursors of the October-November 2022 effusive crisis through seismic and thermal camera measurements. We analyzed the lava overflow on October 9, which was preceded by a crater-rim collapse, and the overflow on November 16. In both cases, seismic precursors anticipating the overflow onset have been observed. The analysis of the seismic and thermal data led to the conclusion that the seismic precursors were caused by an escalating degassing process from the eruptive vent, which climaxed with the overflows. Volcano deformation derived from ground-based InSAR and strainmeter data showed that inflation of the crater area accompanied the escalating degassing process up to the beginning of the lava overflows. The inflation of the crater area was especially evident in the October 9 episode, which also showed a longer seismic precursor compared to the November 16 event (58 and 40 min respectively). These results are important for understanding Stromboli’s eruptive mechanisms and open a perspective for early warning of potentially dangerous phenomena.

## Introduction

**Figure 1:**
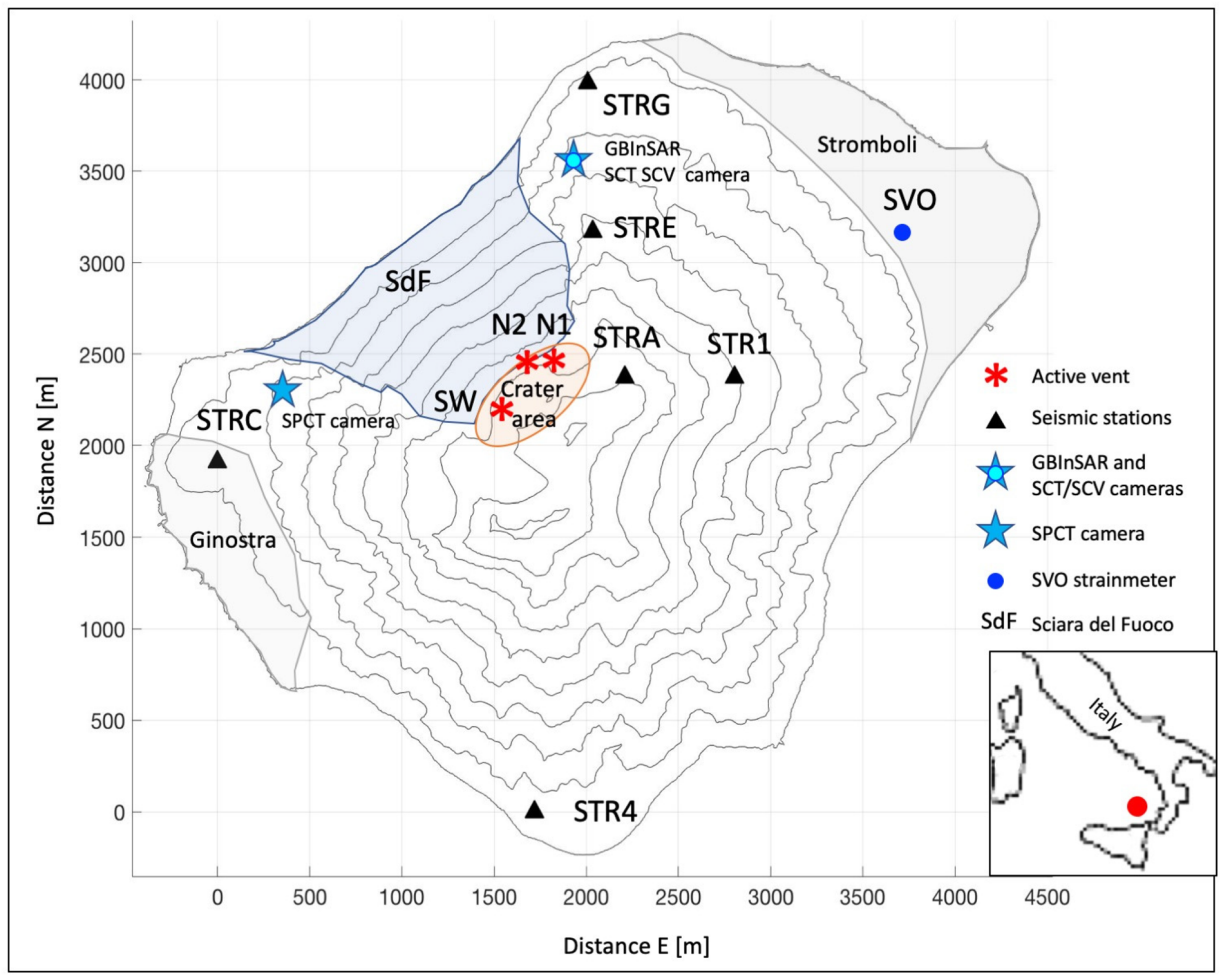
The Stromboli volcano map with the location of the geophysical instruments used for this work. The black triangles are the seismic stations, the blue star with the cyan circle inside indicates the GBInSAR device and the SCT and SCV cameras, the blue star on top of the volcano indicates the SPCT camera, the blue circle marks the position of the SVO strainmeter. The villages of Stromboli and Ginostra, the slope of the Sciara del Fuoco (SdF) and the crater area are shown on the map. The position of the eruptive vent that generated the studied events (N2 vent) is indicated with the N2 red asterisk. N1 red asterisk marks the location of crater N1 and SW red asterisk represents the southwest crater area (SW). The geographical position of Stromboli in the Mediterranean Sea is indicated in the box at the bottom right of the figure (red circle).

Stromboli is an Italian volcano located in the southern Tyrrhenian Sea, north of Sicily (Fig. 1). It is a persistently active volcano producing several hundred Strombolian explosions per day^1^ that originate from different eruptive vents in the crater area (Fig. 1). The morphology of the volcanic edifice is characterized by the imprint of repeated lateral collapses that have particularly affected the north-western side of the island^2^. This slope, called “Sciara del Fuoco”, was shaped by the accumulation of volcanic products erupted by the persistent explosive activity^3,4^, which filled the deep depression created by sector collapses with unstable loose talus^5^. On December 30, 2002, two days after the beginning of an effusive phase, a large landslide broke off the Sciara del Fuoco, partly underwater, and generated a tsunami with waves of about 10 m on the coasts of the island^6,7^. This tsunami caused extensive damage on the island, where two villages are located, Stromboli, in the northern sector, and Ginostra on the southern side (Fig. 1). Fortunately, this tsunami didn’t cause any casualties, but some people were injured. In recent decades Stromboli showed an increase in eruptive activity and produced dangerous phenomena such as lava flows, paroxysms^8^, pyroclastic flows, major explosions, and crater collapses^1,9–12^. The main effusive phases occurred in the years 2002-2003^13–15^, 2007^16^, 2014^10^, and 2019^8^. In addition to these effusive phases, minor lava overflows (hereinafter referred to as overflows) often occurred (at least 78 from September 2008^4,12,17,18^) which have affected the morphology of the Sciara del Fuoco^1,11,12,18^. Although the overflows are generally short-lived events, they can destabilize the slope of the Sciara del Fuoco, especially when they reach the sea rapidly. In some cases, the overflows are preceded by the collapse of the eruptive crater rim, as happened in March-April 2020^11^, on May 19, 2021^12,19^ and on October 9, 2022. In general, the collapse of a portion of the summit crater causes a Pyroclastic Density Current (PDC) on the Sciara del Fuoco, which accelerates along the slope with speeds up to $\sim 40$ m/s and sometimes expands over the sea for tens to several hundred meters^10,11^. This process is potentially tsunamigenic, and even when it does not trigger major landslides, it can cause anomalous waves of modest magnitude. The summit crater collapses because of magma fingering within the poorly welded spatter comprising the summit cinder cone^20^. This process is the result either of ejecta rapid accumulation around the erupting vent, which grows an unstable structure prone to fail^21^, or it is caused by the rise of magma level within the crater^10^. Once the crater fails and the summit cone is breached, it takes several months before the collapsed flank is built up again. Thus, generally, summit crater failures do not recur in sequence, because some time is required for the summit to revert to the highly unstable conditions leading to the collapse episodes.

We selected two overflow events as case studies in which the precursors are clearly evident, allowing us to characterize them. The first event occurred on October 9, 2022, and was characterized by a crater collapse resulting in PDC, which happened before the lava overflowed the N2 crater rim (marked with a red asterisk in Fig. 1). The second episode under analysis occurred from the same vent on November 16, 2022, and did not show any crater collapse. By examining seismic, geodetic, and thermal camera data, we identified medium-term precursors of these overflows. The data, the results obtained and the analysis methods are described below.

## Results

### Eruptive activity on October 9 and November 16, 2022, based on analysis of video camera recordings

The eruptive activity on October 9 was observed and described using the SCT, SCV and SPCT cameras, which provide a side view of the crater terrace from the NNE (SCT, SCV) and the SSW (SPCT), respectively (Fig. 1). The thermal camera SCT offered the best view and was used for a semi-quantitative analysis. A total of 7 vents were observed to erupt on October 9, the early morning. They can be grouped into two crater areas: northeast (N1 and N2 in Fig. 1) and central-southwestern (SW in Fig. 1). Such a high number of small vents is typically associated with a magma level that is not very shallow within the conduit^22^, estimated at few tens of meters depth. The eruptive activity consisted of mild spattering (with heights of the ejecta mostly less than 10 m) but with a high frequency from two vents within N2 crater. These vents exhibited synchronized increasing and decreasing phases every 2–3 h, where only puffing was observed during the lower phases. Strombolian explosions occurred from SW and N1 vents, on average 2–3 times per hour, with the most powerful explosions observed at N1, reaching ejecta heights up to $\sim 150$ m. After 06:24 UTC, the spattering activity from the two vents within N2 increased significantly, causing fallout along the upper Sciara del Fuoco (SdF). The height of the ejecta reached a few tens of meters, and around 07:20, the two jets from the N2 vents merged, likely due to failure of the septum which had separated them. The height of the ejecta further increased, and at 07:22 the northern flank of the N2 crater collapsed, resulting in an overflow from the breached crater rim and a pyroclastic density current. The PDC rapidly spread along the SdF and reached the sea in 28 s, covering a distance of 1130 m at an average speed of $\sim 40$ m/s. The PDC continued to spread on the sea surface for approximately 500 m, similar to previous occurrences documented in the past^1,10,19^. Several pulses of lava overflows were visible from the monitoring cameras, occurring at the breached crater rim approximately every 20 s and spreading on the surface of the well-fed lava flow until 07:32, with a speed of 0.5–2.0 m/s. At 07:44 the lava flow front reached the coast, as observed from the SPCT thermal camera (Fig. 1). The lava overflow drained the upper conduit, and the explosive activity from the summit craters ceased until 07:49 when a new explosion occurred from N1. Spattering within N2 resumed at around 08:00, originating from the previous joined vents, which were now located at a lower elevation within the notch resulting from the failed crater flank. Mild explosions spread spatters in a hemisphere of approximately 30 m radius around the vent, suggesting that magma level was at the crater rim. These explosions occurred while lava was flowing from the breached crater rim and along the SdF. Spattering from the N2 vent and lava flow output along the SdF continued, accompanied by occasional Strombolian explosions from N1 and SW. The explosions became more powerful after 16:00, both in terms of higher frequency of the spattering and of ejecta height (up to $\sim 30$ m). Several smaller crater failures occurred at 11:03 and from 13:39 until midnight originating at the north crater rim, which triggered PDCs spreading above and along the north and south sides of the still well-fed lava flow. The PDCs reached the coast and spread on the sea surface for several hundred meters. After 17:30 the spattering from the N2 vent and the lava flow output rate decreased, and explosions from N2 passed to puffing after 19:30. The lava flow output rate gradually decreased over time and eventually ceased on October 14 (INGV Weekly Report 42/2022, last accessed on 19 May 2023; https://www.ct.ingv.it/index.php/monitoraggio-e-sorveglianza/prodotti-del-monitoraggio/bollettini-settimanali-multidisciplinari).

The episode on November 16 began with intense degassing and spattering from two vents within the N2 notch that had collapsed on October 9, resulting in ejecta reaching a height of a few tens of meters. Powerful explosions from N1 and SW were also recorded, reaching heights approximately 250 m. An additional third degassing vent was located on the north flank of the N2 crater rim in the upper SdF, where the effusive vent of the previous episode has opened. This vent produced degassing and spattering, with ejecta reaching heights of a few tens of meters. Compared to the October 9 episode, on November 16 the magma level was at the crater rim from the beginning of the day, thus causing an abundant spatter fallout in the upper SdF from all erupting vents. At 05:38, the spattering activity from N2 rapidly increased in frequency and ejecta height, and caused abundant spatter accumulation on the summit crater, NE flank. At approximately 06:18, an overflow started from crater north rim. It expanded for $\sim 300$ m on the upper SdF until 07:00 and showed only minor widening until around 10:30. This episode did not produce any PDC, but only minor rockfalls detaching from the lava flow front. The lava flow output was less significant than in the previous event: indeed, with a length of only 300 m, it did not reach the coast, which is located approximately 1.3 km from the N2 vent.

### Escalating degassing processes tracked through seismic and thermal observations

**Figure 2:**
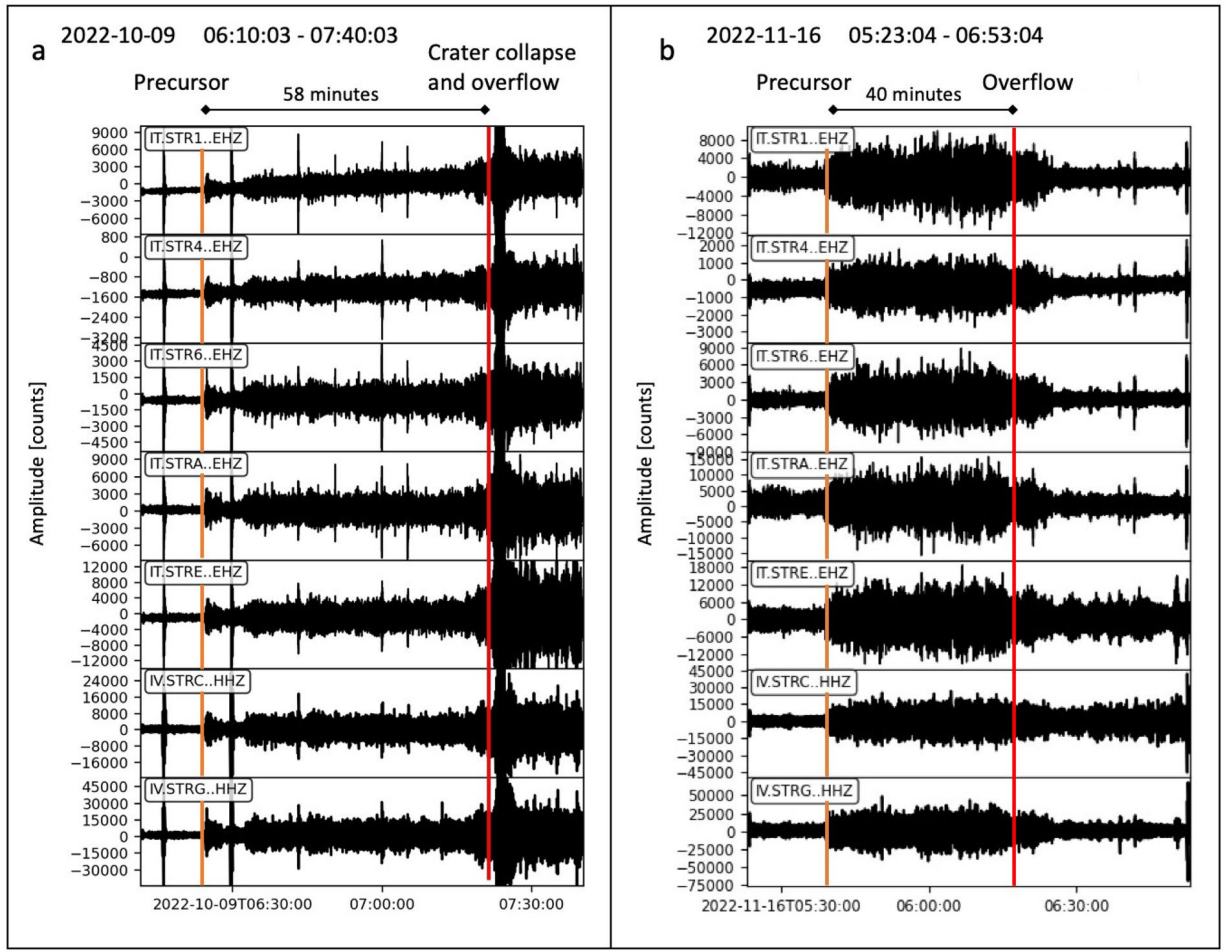
Vertical components of the seismic records of the October 9 (**a**) and November 16 (**b**) 2022 events. The orange lines mark the onset of the seismic precursor, the red lines mark the onset of the overflow. For the location of the stations (rectangle at the upper left of each frame), refer to Fig. 1.

We analyzed the data from the broadband seismic stations installed near the craters, namely STRA (approximately 500 m from the N2 vent), and at Ginostra, namely STRC (approximately 1800 m from the N2 vent), south of the crater area (Fig. 1). The data collected on October 9 and November 16, 2022, revealed an increase in seismic amplitude preceding the onset of the overflows. This increase was observed at all seismic stations (Fig. 2). On October 9, the overflow was preceded by the crater collapse, resulting in a high-amplitude landslide seismic signal (Fig. 2a). In contrast, on November 16, no crater collapse occurred, and the amplitude of the seismic signal decreased after the overflow began.

**Figure 3:**
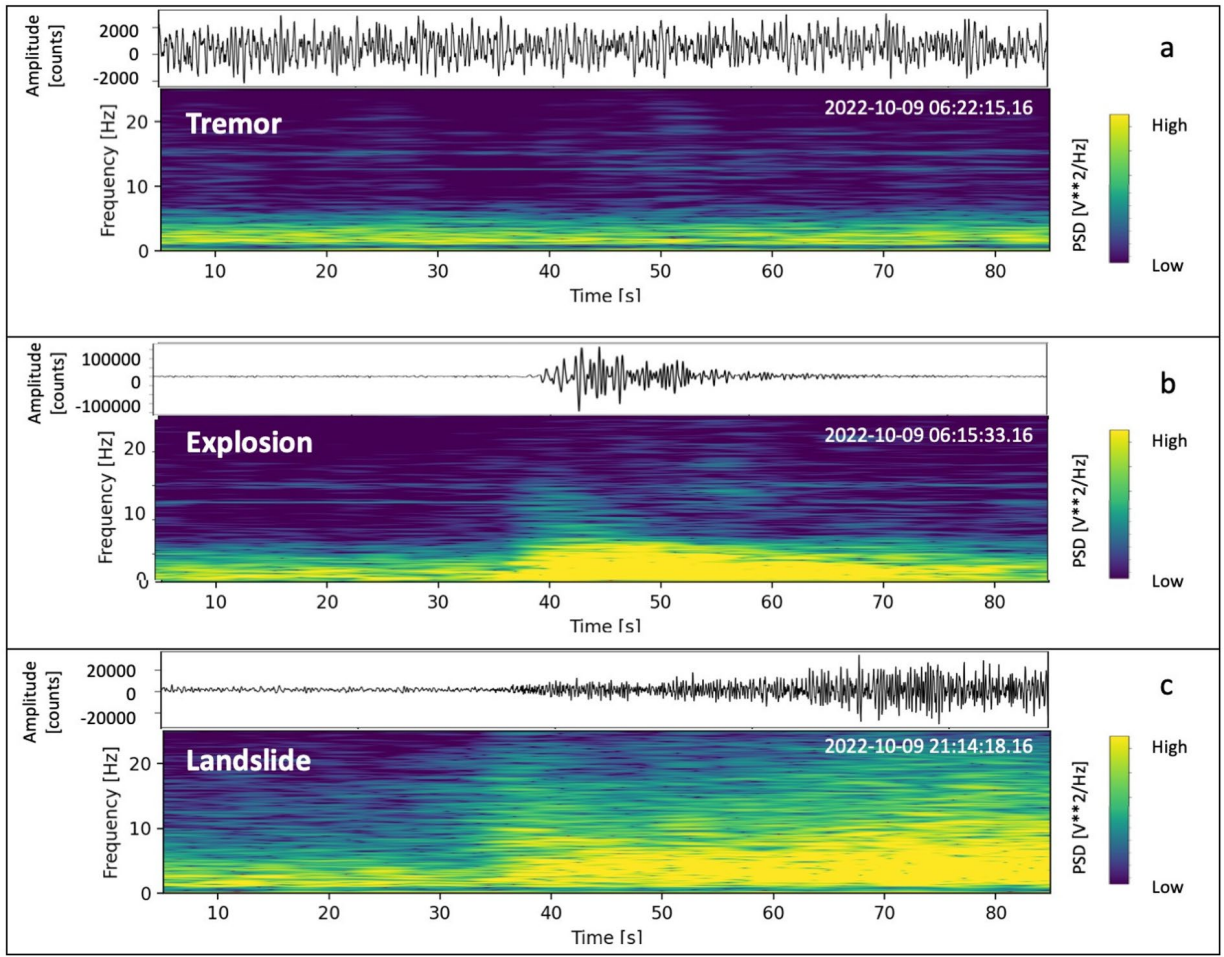
Seismograms and spectrograms of volcanic tremor (**a**), an explosion (**b**) and a landslide (**c**) recorded by the STRC station (component N). The three recordings have a duration of 85 s.

To understand the nature of the seismic signal recorded prior to the overflows (hereinafter referred to as seismic precursor), we first considered what is known about Stromboli’s seismc wave field from previous studies^23–26^. Stromboli is known to exhibit a dominant volcanic tremor in its seismic wave field, which is typically observed in open conduit volcanoes like Yasur, Etna, Villarrica^27–29^. The volcanic tremor is a persistent signal at Stromboli. Ripepe et al. (1996)^30^ proposed that the volcanic tremor at Stromboli was generated by continuous outbursting of small gas bubbles in the upper part of the magmatic column. They based their hypothesis on the analysis of seismic, infrasonic and camera recordings collected at a small distance from the active vents. Chouet et al. (1997)^31^ suggested that the persistent volcanic tremor at Stromboli originated from the collective oscillations of bubbles ascending in a degassing fluid column through the magma conduit. They measured the slowness and back-azimuth of the Stromboli volcanic tremor using seismic arrays installed in the Labronzo area, and determined that the tremor sources were located beneath the summit crater at depths shallower than 200 m. This study highlighted that the signals of tremor and explosions were both dominated by shear horizontal (SH) waves, and the wave field consisted of body and surface waves associated with topography, structure, and source properties. Numerous other studies have been conducted on the volcanic tremor at Stromboli (e.g. Refs.^32–34^). Among them, De Lauro et al. (2006)^35^ analyzed the tremor in the 0.1–0.5 Hz trequency band (referred to as “Stromboli VLP tremor”), and found that it was radially polarized and its source was located in the crater area. Other signals that characterize the Stromboli seismic wave field are the explosion-quakes, which are generated by the ordinary eruptive activity in the crater area, and the signals produced by landslides on the Sciara del Fuoco. The tremor typically exhibits frequency content within the 1-3 Hz range (Fig. 3a). The signals generated by the explosions are characterized by Very Long Period (VLP) events with frequencies in the 0.05–0.5 Hz range and mostly have a spectral content at higher frequencies between 1 and 7 Hz (Fig. 3c). Landslides generate signals with higher frequencies, reaching up to 20 Hz (Fig. 3c).

**Figure 4:**
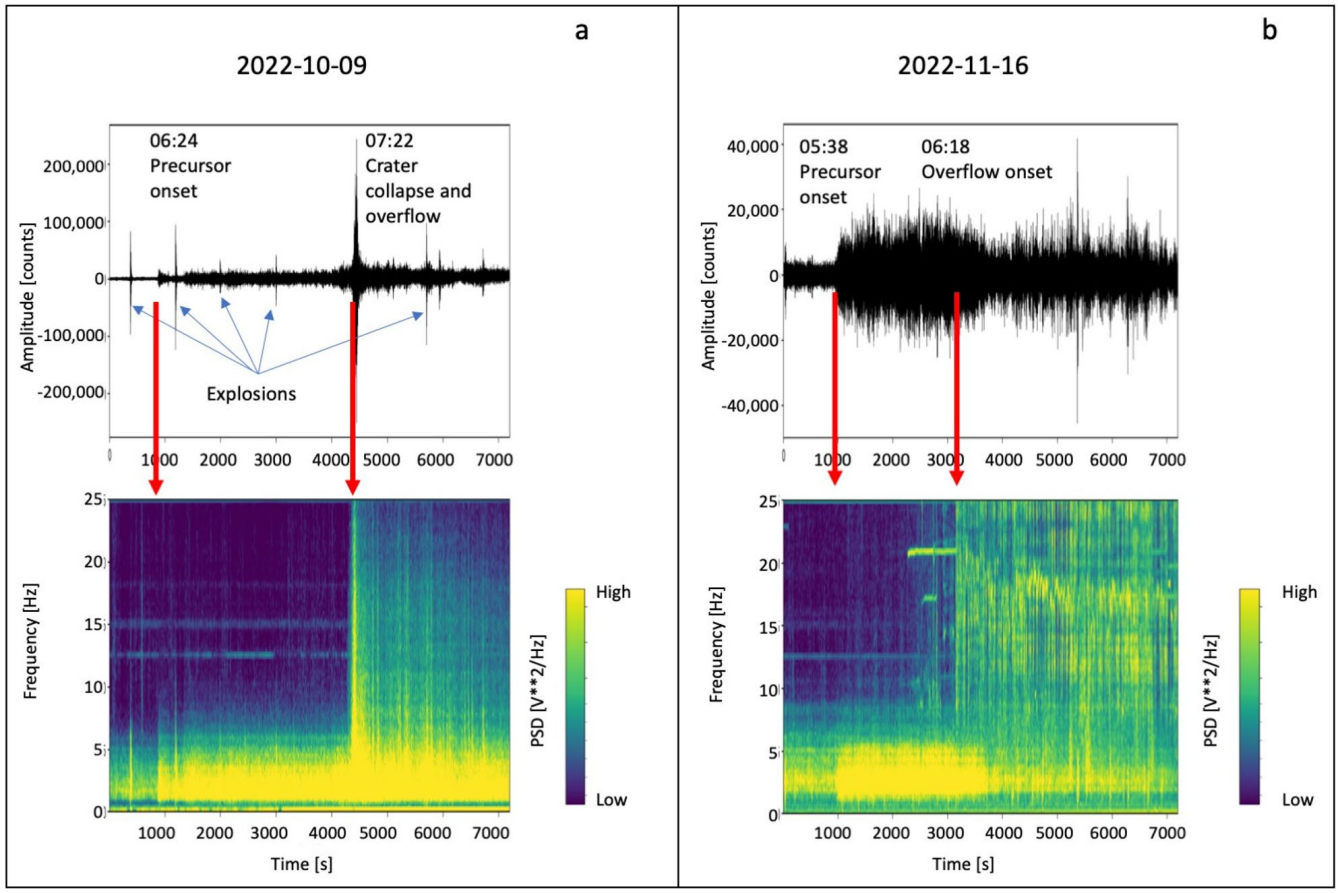
Seismograms and spectrograms of two-hour recording, relevant to the overflows on October 9 (**a**) and November 16 (**b**), 2022. The panel (**a**) time window, relevant to the October 9 event, starts at 6:10 (UTC) and the duration is 7200 s (2 h). The time window of panel (**b**), relating to the November 16 event, starts at 5:23 (UTC) (duration 7200 s = 2 h). The red arrows mark the onsets of the seismic precursors and the onset of the overflows, respectively.

**Figure 5:**
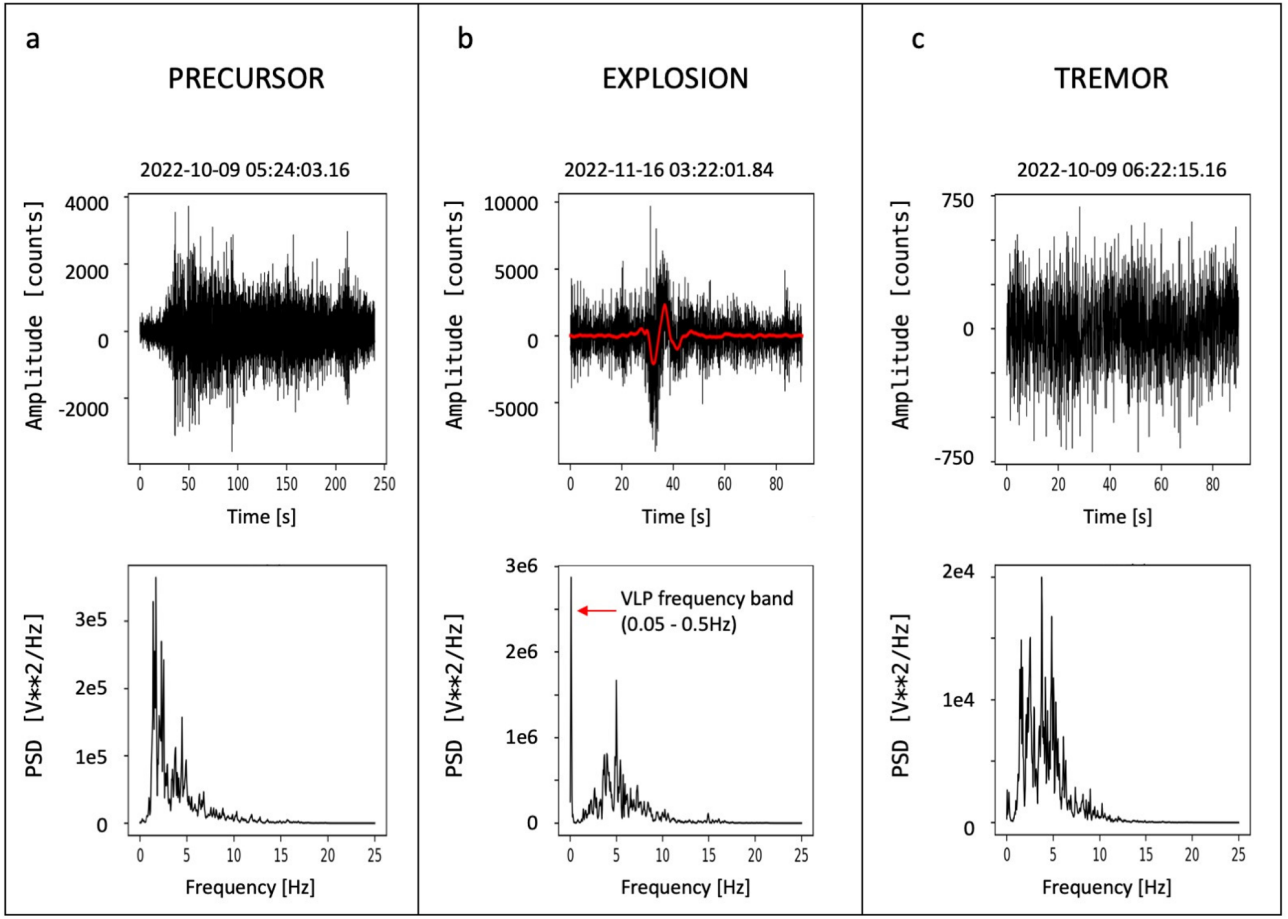
Comparison between the spectral content of the seismic precursor of the October 9 2022 episode (**a**) and a signal generated by an explosion (**b**). Panel (**a**) shows the seismogram and power spectral density of the seismic precursor onset. Panel (**b**) shows the seismogram and power spectral density of the explosion signal. Note the peak due to the VLP component. The filtered signal in the VLP band (0.05–0.5 Hz) is shown in red on the seismogram. Panel (**c**) shows the seismogram and power spectral density of the tremor recording.

To gain insights on the source processes generating the progressive increase in seismic amplitude in the two overflows under analysis, we calculated the spectrogram of the two sequences (Fig. 4), and conducted a detailed comparison between the spectral characteristics of the seismic precursor and those of the explosion and tremor recordings. It was found that the spectra of the three types of signals significantly overlap (Fig. 5). Figure 4 illustrates that a signal with a frequency content compatible with volcanic tremor predominates before the seismic precursor onset. In both episodes, the precursor is defined as signal between the tremor and the onset of the overflow. It exhibits a frequency content similar to that of the explosion-quakes (which also includes the frequencies typical of volcanic tremor), but without VLP pulses. During this interval, the signal is continuous (Figs. 4, 5) and the VLPs, typically associated with the explosion-quake signals (Fig. 5), are only recorded in coincidence with some explosion. In the October 9 episode, the collapse of the crater generated a significant landslide signal characterized by higher frequencies compared to the other seismic signals typically observed at Stromboli (Fig. 4a). Even after the collapse, smaller-amplitude landslide signals persisted due to the continuous rolling of blocks from the lava flow front and along the slope, generating a landslide seismic signal^26^ (Fig. 4a). On November 16, the overflow was not preceded by a crater collapse. However, also in this case, the rolling blocks from the lava front generated landslide-like signals identifiable in the spectrogram high frequencies (Fig. 4b). The seismic amplitude in the frequency band typical of landslide signals (>10 Hz) is relativey small, and therefore, this component is not dominant in the wave field produced by the November 16 episode.

**Figure 6:**
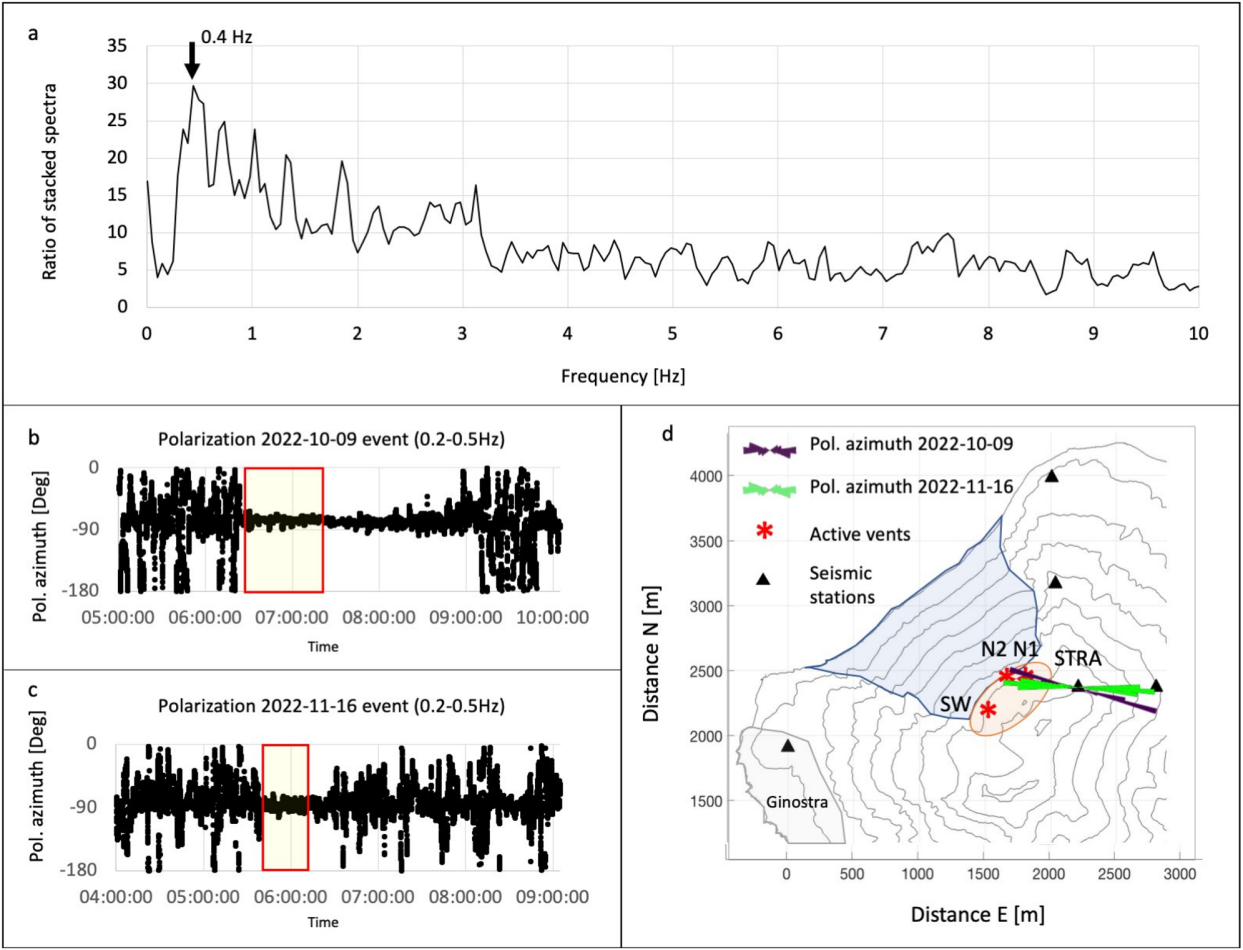
(**a**) Ratio of stacked spectra of seismic precursor (15 windows of 1-minute length) and tremor (20 windows of 1-minute length). The maximum of the stacked spectra ratio corresponds to the 0.4 Hz frequency. (**b**) Azimuth of the seismic signal polarization of the STRA station in the frequency band 0.2–0.5 Hz, relevant for the overflow episode of 9 October 2022 (from 5:00 to 10:00). The rectangle with the red border highlights the segment used for the rose diagram of panel d (purple rose). (**c**) Azimuth of the seismic signal polarization of the STRA station in the 0.2-0.5 Hz frequency band, related to the overflow episode of 16 November 2022 (from 4:00 to 9:00). The rectangle with the red border highlights the segment used for the rose diagram of panel d (green rose). (**d**) Rose diagrams of the polarization azimuth of the two overflow episodes superimposed on the Stromboli map. The positions of the STRA station and the active vents are shown on the map. The thermal precursor is located on vent N2.

**Figure 7:**
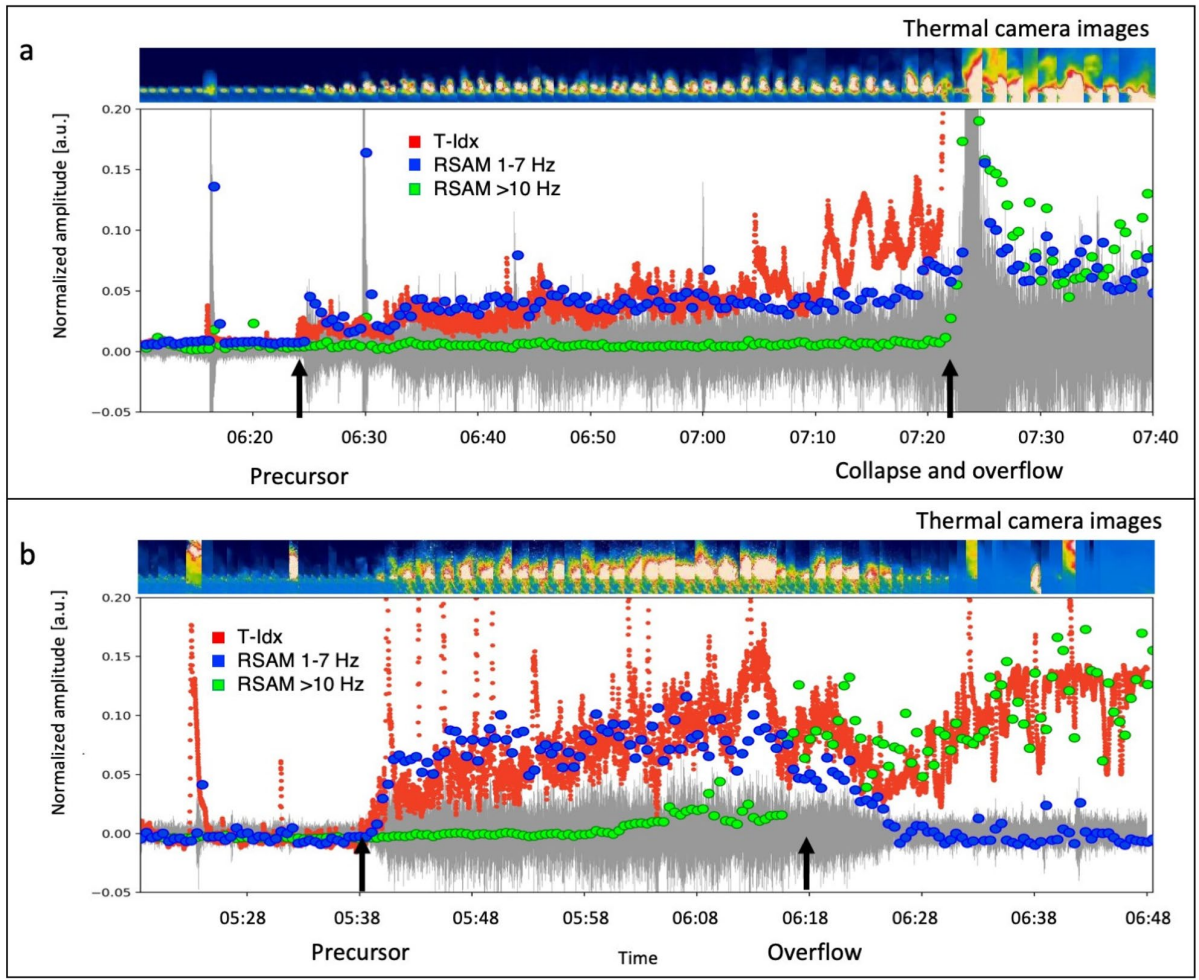
Comparison of seismic parameters with the T-Idx for the overflow on October 9, start time 06:10:00.00 UTC (**a**) and November 16, start time 05:24:00.00 UTC (**b**). The green dots represent the seismic amplitude in the $>10$ Hz band, the blue dots the seismic amplitude in the 1–7 Hz band and the red dots indicate the T-Idx. For both panels, the seismogram is shown in grey in the background. A sequence of images from the thermal camera, which provides a visual representation of the phenomenon, is shown at the top of each panel.

From a seismological perspective, the seismic precursor can be classified as a type of tremor. In order to examine any distinctions between the seismic precursor and the typical volcanic tremor observed at Stromboli, a more detailed spectral analysis of both signals was conducted. We stacked the spectra of 1-minute windows of recording for the seismic precursor and the tremor in both case studies. Subsequently, the ratio between the stacked spectra of the seismic precursor and tremor was calculated (additional analysis details are provided in the Supplementary Material). From the stacked spectra ratio, it can be observed that in the October 9 episode, the seismic precursor exhibited significantly higher amplitude compared to the ordinary tremor, particularly in a narrow frequency band around 0.4 Hz (Fig. 6a). To further investigate this frequency band, we filtered the seismic data of the three components of the STRA station (the closest one to the active vents) by applying a narrow bandpass filter around 0.4 Hz (0.2–0.5 Hz). The data filtering was performed using a two-pole, zero phase shift Butterworth-bandpass filter. Subsequently, we computed the polarization of the STRA seismic signal. For the signal analysis, ObsPy^36–38^, an open-source Python system for processing seismological data, was employed. Our findings reveal that the polarization azimuth mostly aligns with the direction of the active vent (N2) when the seismic precursor emerges. This occurs in both episodes (Fig. 6b–d). The mean incidence angle of polarization in the analyzed time intervals depicted in the rose diagrams of Figure 7d (panel b: 6:25–7:20 UTC, October 9, 2022, and panel c: 5:39–6:10 UTC, November,16, 2022), was approximately 10 degrees with a dip towards the N2 vent during the October 9 event, and about 6 degrees with the same dip, for the November 16 event.

By visually analyzing the images captured by the SCT camera (Fig. 1) during the October 9 overflow, we observed a correlation between the seismic precursor and an intense phase of degassing and the spattering from the N2 vent located in the northeastern sector of the crater area (Fig. 1). The outgassing phase persisted continuously until the collapse of the crater (58 min after the seismic precursor onset), which was immediately followed by the overflow. Thus, the continuous bursting of bubbles at the vent was sustained by the increasing outgassing activity over time. A similar degassing phase, associated with the seismic precursor, was observed before the overflow on November 16, 2022, based on the analysis of thermal camera images. Notably, at the same moment when the seismic amplitude increase began at 5:38, an intense outgassing and spattering activity was observed at the N2 vent. The overflow from the N2 vent started at 6:18, 40 min after the seismic precursor onset, and the concurrent onset of intense outgassing and spattering activity at vent N2.

To compare the camera recordings with the seismic data, we carried out a basic image processing (see the Methods section for details). This method allowed us to calculate a parameter, which we named Temperature index (T-Idx). The T-Idx is proportional to the temperature of a $60\times 60$ pixel sector framing the eruptive vent (N2). Figure 7 shows the comparison between the T-Idx (represented by red dots) and the seismic amplitude, expressed as RSAM^39^, calculated in two frequency bands: 1–7 Hz (blue dots) and $>10$ Hz (green dots). The 1–7 Hz frequency band is sensitive to the seismic precursor, and encompasses the characteristic frequencies of volcanic tremor and Strombolian activity. The > 10 Hz frequency band is particularly sensitive to the rolling of blocks on the Sciara del Fuoco, thereby highlighting landslides and block detachment from the lava overflow front. The T-Idx, which is sensitive to the continuous bursting activity associated with the escalating outgassing from the N2 crater, exhibits a temporal evolution in good agreement with the seismic amplitude in the precursor frequency band (1–7 Hz). Both parameters show a sharp increase starting 58 and 40 min before the October 9 and November 16 events, respectively. When the seismic precursor emerges, the RSAM 1–7 Hz abruptly increases by a factor of 4.9 for the October 9 event and 3.3 for the November 16 event. Moreover, both the RSAM 1–7 Hz and the T-Idx show an increasing trend until the overflows begin. We calculated the correlation coefficients between of the RSAM 1–7 Hz and the T-Idx time series (within a one-hour segment containing the onset of the seismic and thermal precursors), obtaining a correlation coefficient of 0.88 for the October 9 episode and 0.93 for the November 16, 2022 episode (refer to the Supplementary Material). Conversely, the seismic amplitude in the > 10 Hz frequency band, associated with the landslides on the Sciara del Fuoco, only increases after the crater collapse and the overflow in the October 9 event, and after the overflow onset in the November 16 event. This indicates that the seismic precursor cannot be attributed to landslides on the slope of the Sciara del Fuoco.

### Ground deformation before overflows from GBInSAR measurements

**Figure 8:**
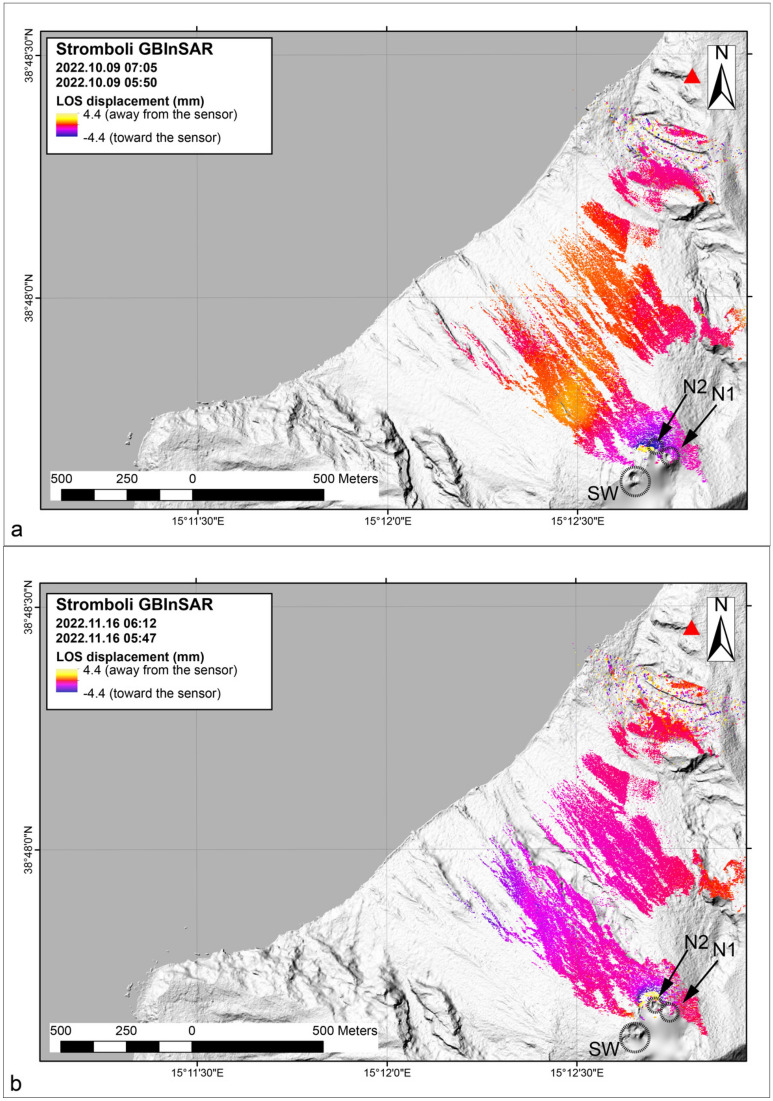
The interferograms relating to the overflow events on October 9 (**a**) and November 16 (**b**) 2022. The active vents are indicated with N1, N2 and SW.

We analyzed the ground deformation data by studying the line-of-sight displacement measured using a Ground-Based Interferometric Synthetic Aperture Radar (GBInSAR) device, which is installed at Stromboli. The device monitors the Sciara del Fuoco and the crater terrace. Over the long term (one week before the event), the episode on October 9 showed a clear increase in the displacement of the ground toward the device, indicating the inflation of the crater terrace^40^. In contrast, the overflow on November 16 did not show significant changes over the long term^41^. By comparing the seismic and thermal parameters with the deformation data obtained from the GBInSAR measurements, we found that the phase of escalating outgassing activity that preceded the two overflows was characterized by an inflation of the crater area, with maximum velocity in the line of sight of about 8.8 mm/hour for both events. The GBInSAR field of view (Fig. 8) does not allow for an accurate reconstruction of the source volume variations. However, the interferograms indicate that the zone affected by the deformation is the same in both episodes and corresponds to the volcano cone summit (Fig. 8).

### Deformation through strainmeter measurements

**Figure 9:**
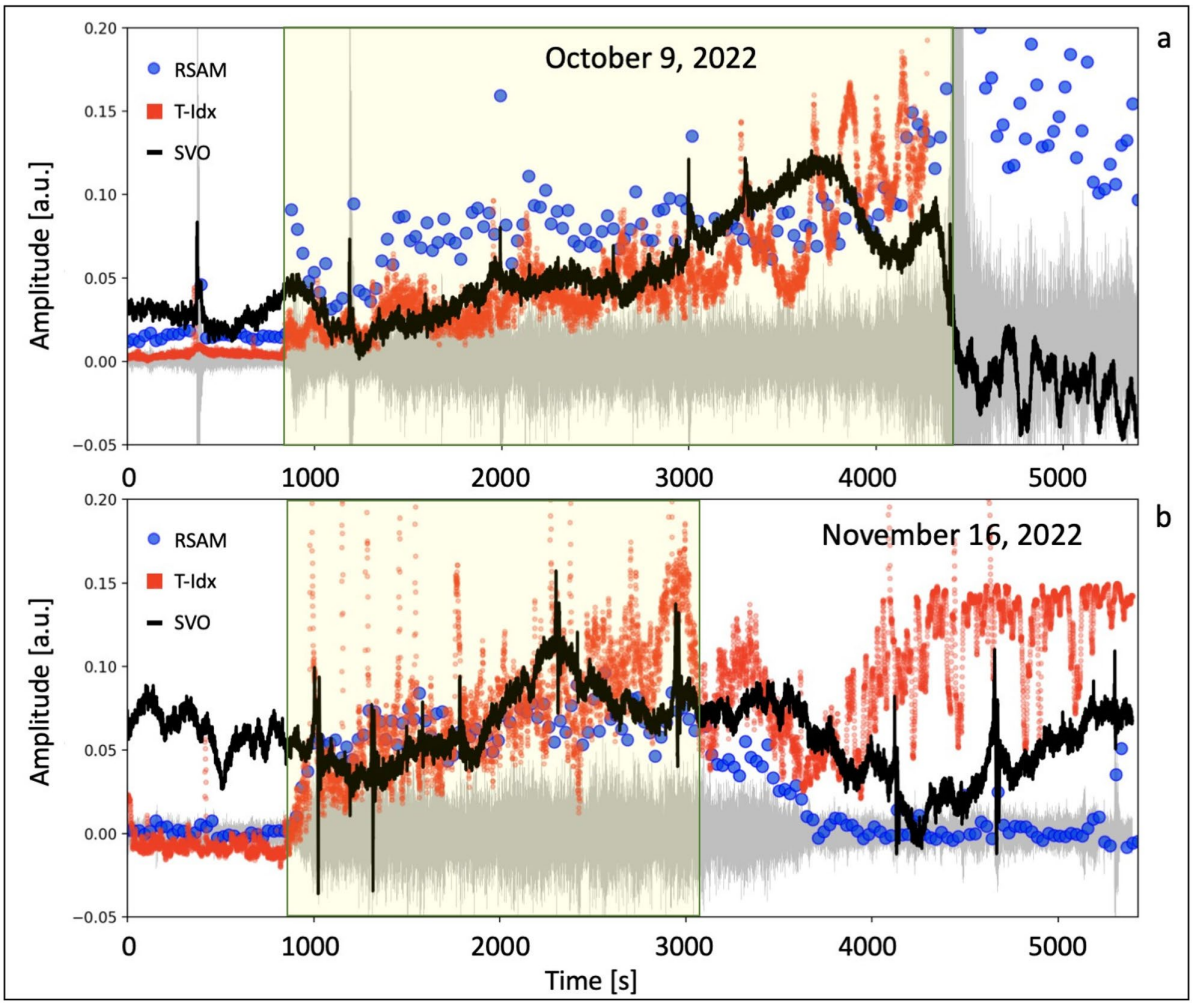
Comparison between the seismic precursor (RSAM of the northern component signal of the STRC station, filtered in the 1–7 Hz frequency band) indicated by the blue dots, the T-Idx temperature index, marked by the red dots, and the SVO strainmeter signal (black line), filtered in the 0.00014–0.5 Hz frequency band, for the overflow on October 9 (**a**) and November 16 (**b**), 2022. The yellow rectangles indicate the time intervals between the onset of the seismic and thermal precursors and the onset of the overflows. These timeslots are 58 min, starting at 06:24 UTC and 40 min, starting at 05:38 UTC, for the events on October 9 and November 16, 2022, respectively. The amplitudes are normalized with the addition of appropriate offsets for the legibility of the graph.

To gain further insight into the temporal evolution of Stromboli’s elastic deformation occurring just before the two overflow episodes under study, data from the Sacks-Evertson borehole strainmeter^8,42–44^ located at the the SVO (blue circle in Fig. 1) were examined. The strainmeter signals have a sampling rate of 50 sps. We filtered the strainmeter signal in the frequency band between 2 s and 2 h (0.00014–0.5 Hz), and compared it to the seismic and thermal parameters recorded during the October 9 (Fig. 9a) and November 16 (Fig. 9b) overflow events. Figure 9 shows that both overflows were preceded by a strain transient that well correlates with the evolution of the seismic amplitude in the precursor frequency band (1–7 Hz) and with the T-Idx obtained from the thermal camera measurements. In addition, the October 9 episode exhibited a stronger increase in strain before the overflow, compared to the other event.

## Discussion

The results of this study demonstrate that the overflow events in October and November 2022, selected as case studies to analyze the precursors of effusive activity on Stromboli, were clearly preceded by seismic, thermal and geodetic precursors. The earliest and most prominent precursors were the seismic and thermal signals, which occurred 58 and 40 min before the overflows in October and November 2022, respectively. Visual analysis of the camera recordings revealed that the thermal precursor was due to the sudden appearance of intense and continuous outgassing, accompanied by spattering, at vent N2 (Figs. 1 and 6). The seismic precursor, technically classified as a tremor, exhibited subtle differences with respect to the ordinary Stromboli tremor, as highlighted through spectral and polarization analysis (see Supplementary Material). In particular, the frequency band between 0.2 and 0.5 Hz played a more significant role in the seismic precursor than it does in ordinary tremor recordings. The polarization analysis of this seismic component provided information on the source location, indicating a clear orientation towards the N2 vent with small angles of incidence (averaging 10 degrees for the October episode and 6 degrees for the November episode) directly pointing to the vent. These findings suggest that the intense outgassing process, which also generates the thermal precursor, is the source process responsible for the seismic precursor that is localized at the N2 vent. The two events under study also exhibited some differences: the overflow on October 9 was characterized by a crater-rim collapse resulting in a PDC that rapidly spread over the Sciara del Fuoco and extended approximately 500 m over the sea. In contrast, the November 16 event was preceded and accompanied by an intense explosive activity. This activity can be well characterized by the VLP size parameter which quantifies the magnitude of the VLP seismic signals generated by the explosions^8^. In the 2 h preceding the overflow events, this parameter exhibited average values (in counts) of 519 and 1788 for the October 9 and November 16 events, respectively, resulting in a ratio of 3.4. However, in both cases the overflows were preceded by a progressive increase in sustained bursting activity from the eruptive vent, driven by the intense outgassing associated to magma ascent in the shallow conduit. The results obtained provide a consistent understanding of the overflow events: we interpret the sudden appearance of the seismic and thermal precursors of the October-November 2022 Stromboli overflows as a discrete episodes of increased outgassing linked to the ascent of a magma batch. Furthermore, the GBInSAR and strainmeter deformation measurements indicate that, the deformation before the October 9 overflow, which started with the crater collapse, was higher than the deformation observed before the overflow on November 16. This suggests that deformation measurements, when compared to seismic and thermal data, could indicate which overflow episodes generate the crater rim collapse which do not.

This study confirms that seismic measurements are highly sensitive to outgassing processes, as research in this field had already extensively highlighted^39,43,45–54^. In the case of Stromboli, which is a natural laboratory due to its persistent activity and easy accessibility, seismic methods combined with thermal and deformation measurements are making it possible to recognize precursors to potentially hazardous eruptive phenomena, such as paroxysms^43,55^ and lava overflows^11,55^. These phenomena typically exhibit variations in the degassing processes preceding them, which can be detected through seismic data analysis, and interpreted by comparing seismic parameters with camera and deformation data. This was also observed in Giudicepietro et al. (2022)^55^, where an anomalous period dominated by gas explosions that had begun more than a month before the 2019 paroxysmal phase at Stromboli was discovered through machine learning analysis of seismic data. However, Stromboli experiences continuous changes in the summit area’s morphology^56^, resulting in variations of the active vents and the slope of the Sciara del Fuoco^4^. These factors can influence the dynamics of the possible precursors. The properties of the seismic signals generated by outgassing processes are influenced by various factors, including magma viscosity, gas flux velocity, and conduit roughness^55,57–59^. Standardizing monitoring based on specific parameter thresholds is therefore challenging. Consequently, the key to generalizing the results of this study is to focus on changes in the style of outgassing processes (and eruptive style of the persistent activity, in general) that typically precede potentially dangerous variations in the eruptive activity.

An interval of some tens of minutes provides an appropriate timeframe to implement actions ensuring the safety of people on the island, such as relocating individuals from the Sciara del Fuoco area. Therefore, this study can have positive implications for enhancing the monitoring system and can serve as inspiration for designing an effective early warning system. Future developments could involve equipping the monitoring system with tools for real-time automatic recognition of the precursors identified in this study. Furthermore, considering that precursors exhibit slight differences even between events occurring within a month approximately, the monitoring system could benefit from tools capable of detecting more general variations in the properties of seismic and thermal signals, since these could be precursors to anomalous activity. State-of-the-art machine learning methods could effectively approach this type of problem.

In general, the study of precursors and subsequent improvements in monitoring systems require multidisciplinary data. Seismic measurements typically show an early response to changes in a volcano’s activity style, as exemplified in our case where the seismic precursor clearly coincided with degassing variations. Thermal measurements obtained from permanent video cameras also exhibit distinct variations when visibility conditions are favorable, contributing to the accurate interpretation of the seismic precursor signals. Deformation measurements using strainmeters and GBInSAR provide information about rising magma in the shallow plumbing system and identify portions of the volcanic edifice subject to detectable surface deformations^60^. Therefore, multiparametric observations not only aid in identifying precursors but also facilitate the interpretation of the underlying processes, albeit at a conceptual level.

## Methods

Most of the seismic stations in the Stromboli network are equipped with Guralp CMG40T (0.016-50 Hz) broadband velocimeters^61^. The signals are recorded using 24-bit digital dataloggers^62^. The sampling rate is 50 samples per second (sps). Recently, the STRC and STRG stations have been upgraded and now are equipped with 3ESPC (0.008-100 Hz) broadband velocimeters. These stations use Affinity Guralp recorders for data acquisition, with a sampling rate of 100 sps (Fig. 1). The seismic data is transmitted in real time at Osservatorio Etneo (INGV, Catania) and Osservatorio Vesuviano (INGV, Napoli). In our analysis, the time series of the seismic amplitude, represented as the RSAM, were calcuated in different frequency bands. Specifically, we focused on the frequency band 1–7 Hz, which is characteristic of the seismic precursor identified in this study, and the $>10$ Hz frequency band, which indicates landslides and rolling of blocks from the front of the overflows on the Sciara del Fuoco (Figs. 3, 4 and 5). We filtered the signal using the ObsPy Toolbox^38^. We applied two filters on the raw signal: a two-pole, zero phase shift Butterworth bandpass filter in the band 1–7 Hz, and a Butterworth high-pass filter with a cut-off frequency of 10 Hz. Subsequently, we calculated the RSAM, which stands for Real-time Seismic Amplitude Measurement, according to the classical formulation^39,63^. The RSAM is determined by averaging the absolute values within a sliding window of the signal:

1 $$\begin{aligned} RSAM(iT) \,=\, \frac{1}{T}\, \sum _{t=iT-\frac{T}{2}}^{iT+\frac{T}{2}} |s(t) |\end{aligned},$$

where *T* is the averaging interval (30 s) and *s*(*t*) is the filtered seismic signal. Furthermore, the polarization analysis of the seismic signal using the ObsPy system utilities was performed to characterize the source of the seismic precursor. The analysis is based on the eigenstructure decomposition method^64^. We have identified a narrow frequency band of interest through the ratio of precursor/tremor spectra (see Supplementary Material). Then, we filtered the signals of the 3-component station STRA in this band (0.2–0.5 Hz) and applied the polarization analysis on the daily time series on October 9 and November 16 using a 1-minute sliding window.

To compare the RSAM time series to the camera recordings, a basic image processing was carried out. We analyzed the images from a thermal camera installed at an altitude of 190 m a.s.l. on the northwestern edge of the slope of the Sciara del Fuoco. This thermal camera recorded the two studied episodes (the overflows which occurred on October 9 and November 16, 2022) from the Labronzo area (SCT), this being a particularly favourable point to record the ordinary eruptive activity of the craters, landslides, and overflows along the Sciara del Fuoco. The thermal images are also transmitted in real time to the data centers of the Osservatorio Etneo (INGV, Catania) and Osservatorio Vesuviano (INGV, Napoli). The thermal camera provides images at a rate of 2 frames per second (fps). Each image is a matrix of color values corresponding to the temperature recorded at each pixel. As a first approximation, we assumed a linear relationship between the temperature and the brightness of the image, defined here as the average of the red, green and blue channels. Subsequently, we cropped a portion of the image containing the N2 crater (Fig. 1). This portion is a square sector with dimensions of 60x60 pixels. The target sector was converted into a black and white image and the pixel values were avaraged. Thus, we obtained a parameter which is roughly proportional to the average temperature of the target area. This parameter was named Temperature Index or T-Idx expressed in arbitrary units. At this point, we compared the T-Idx time series to the seismic amplitude time series in the precursors frequency band (1–7 Hz). The comparison shows an agreement between the two values (Fig. 7 and Supplementary Material).

Ground-Based Synthetic Aperture Radar devices, using Interferometric SAR technology (GBInSAR^65^), are remote sensing imaging systems^66,67^ that emit and receive microwave pulses. They repeate this operation by moving real antennas along a rail (track), and the length of this rail determines the cross-range resolution of the acquired images^65,66^. GBInSAR devices allow to measure one-dimensional (1D) ground motion along the sensor line of sight (LOS) direction by exploiting the phase difference between acquisitions with the purpose of deriving information on deformation at the observed scene^68^. The GBInSAR device used in this work is installed on the northern edge of the Sciara del Fuoco (Fig. 1), at an elevation of 190 m above sea level. It operates in the Ku-band (17.0-17.1 mm radar), with a revisiting time of 6–7 min, and performs image averaging over 30 min to increase the signal-to-noise ratio^68^. The stages of creating displacement maps (interferograms or cumulative maps) include a resample operation that returns images with a pixel size of about 2 m $\times$ 2 m along both range and cross-range. A pixel by pixel stacking algorithm allows for the reconstruction of the cumulative displacement, setting a coherence ($>0.7$; see^65^) and a power filter ($>55$ dB^11^), allowing for the tracing displacement time series of selected points (averaged over $5 \times 5$ pixels) with displacement measurement precision of 0.5 mm^69^.

The SVO device is a Sacks-Evertson borehole strainmeter located at the San Vincenzo Observatory in the northeast sector of the island (Fig. 1). It is installed at a depth of about 200 m below ground level. SVO is situated in competent rock below the sea level. The device has a strain resolution of approximately $10^{-11}$. Its distance from the crater region is about 2.4 km. Data are transmitted to the Osservatorio Vesuviano (INGV-Naples) via TCP/IP and are acquired by a 24-bit digital recorder. The signals are recorded at two different sampling rates, namely 1 Hz and 50 Hz. To remove the strong tidal component, the strainmeter data must be filtered in the most suitable frequency band for the analysis. In our case we applied a two-pole, zero phase shift Butterworth-bandpass filter, using the ObsPy system utilities, and filtered the signal between 2 s and 2 h.

## Supplementary Information

Supplementary Information.

## Data Availability

The data time series analyzed in this study are available upon request to Flora Giudicepietro (flora.giudicepietro@ingv.it).
