# Supplementary material for: Seismic and thermal precursors of crater collapses and overflows at Stromboli volcano
Source: Sci Rep. 2023 Jul 10;13:11115. doi: 10.1038/s41598-023-38205-7 (PMC10333280; doi:10.1038/s41598-023-38205-7)
Supplement: Supplementary file 1 — Supplementary Information. [file 41598_2023_38205_MOESM1_ESM.pdf]

Supplementary Information

Seismic and thermal precursors of crater collapses and overflows at Stromboli volcano

Flora Giudicepietro, Sonia Calvari, Walter De Cesare, Bellina Di Lieto, Federico Di Traglia, Antonietta M. Esposito, Massimo Orazi, Pierdomenico Romano, Anna Tramelli, Teresa Nolesini, Nicola Casagli, Pierfrancesco Calabria, Giovanni Macedonio

Spectra stacking and analysis of seismic signal polarization

To better characterize the seismic precursor signal and highlight any differences compared to the ordinary volcanic tremor of Stromboli, we conducted a comparison of the stacked spectra of the two types of signals, for both case studies (October 9 and November 16, 2022). For the October 9 event, we selected 21 one-minute windows (between 7:00 and 7:21 UTC) of precursor signal, and 15 one-minute windows (3:20 to 3:35 UTC) of tremor. These time intervals were carefully selected to ensure they were not affected by significant explosion signals. Then, we computed the spectra of each precursor window and stacked them. We performed the same analysis with the one-minute windows of the tremor signal. Finally, we calculated the ratio between the stacked spectra of the precursor and the stacked spectra of the tremor. We repeated the same analysis for the November 16 event using a precursor signal segment from 6:03 to 6:13 (10 one-minute signal windows) and a tremor segment from 3:23 to 3:40 (17 windows of on- minute of signal). Table S1 shows the summary of the time intervals used for stacking the spectra for the two events.

Table S1

	Tremor [UTC]	Precursor [UTC]
October 9, 2022	3:20-3:35 (15 1-minute windows)	7:00 to 7:21 (21 1-minute windows)
November 16, 2022	3:23-3:40 (17 1-minute windows)	6:03-6:13 (10 1-minute windows)

Figure S1 shows the results of the spectral analysis and the ratios of the stacked spectra for the events of October 9 (Fig. S1c) and November 16, 2022 (Fig. S1f). Figure S1 also displays the most significant peaks of the stacked spectra ratios, which are indicated as n.1 for the October 9 event, and n. 2 for the November 16 event.

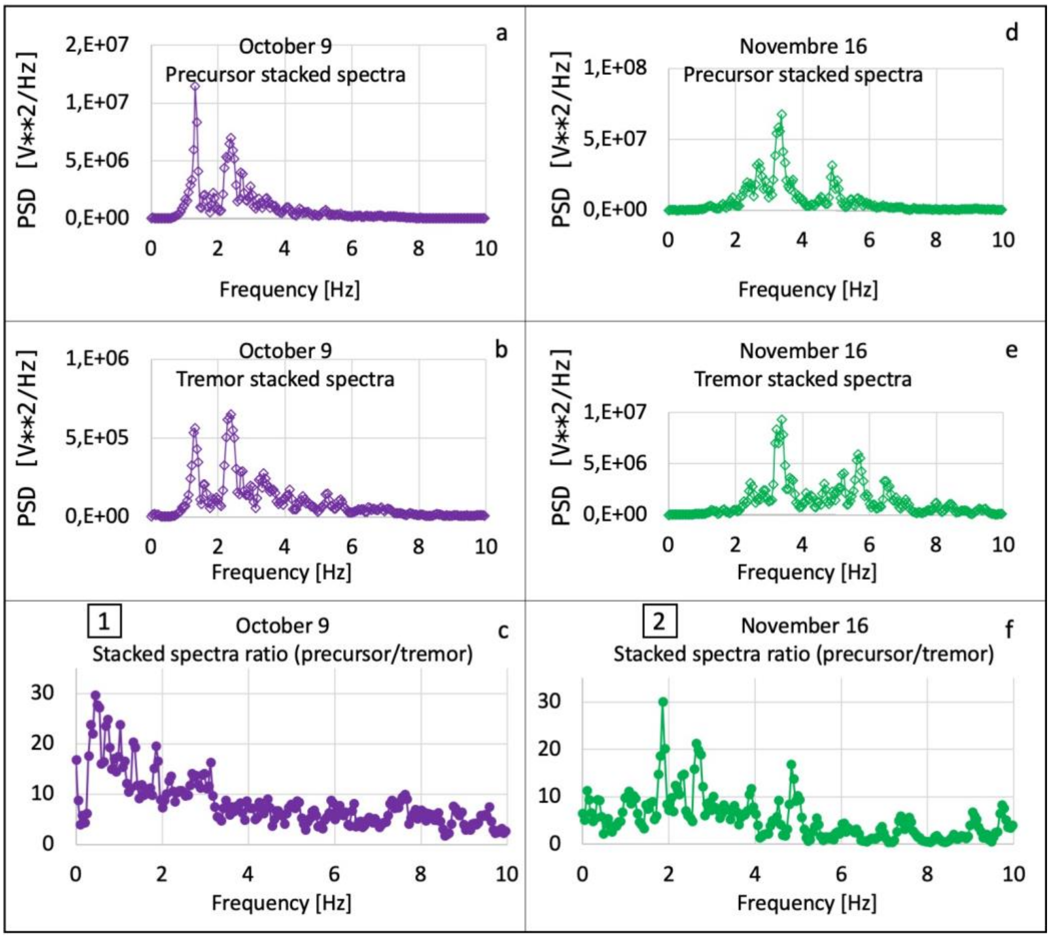

Figure 1 a) Stacked spectra of seismic precursor of the October 9 overflow event (21 windows of 1-minute length). b) Stacked spectra of tremor recorded before the October 9 overflow event (15 windows of 1-minute length). c) Ratio of stacked spectra of seismic precursor and tremor for the October 9 overflow event. d) Stacked spectra of seismic precursor of the November 16 overflow event (10 windows of 1-minute length). e) Stacked spectra of tremor recorded before the November 16 overflow event (17 windows of 1-minute length). f) Ratio of stacked spectra of seismic precursor and tremor for the November 16 overflow even.

The peak relevant to the event on October 9 (n.1 in Figure S1), corresponds to a frequency of 0.43 Hz, whereas the peak relevant to the event on November 16 (n.2 in Figure S1), has a frequency of 1.8 Hz. We filtered the data from the three components of the STRA station (the closest one to the crater area, with a distance of approximately 500 m from the active vents) in narrow frequency bands around the two peaks (0.2-0.5 for peak n.1 and 1.4- 2.3 for peak n.2). Then, we performed the polarization analysis on the filtered signals, using the ObsPy libraries. For this analysis, we adopted a one-minute sliding window.

Figures S2 to S5 present the results of the polarization analysis within these two frequency bands for the two case studies. On the vertical axes of the figures, azimuth ranges between 0° and 180° (from north to east) and 0° and -180° (from north to west). Incidence ranges between 0° (down) to 180° (up). Rectilinearity and planarity values vary between 0 and 1.

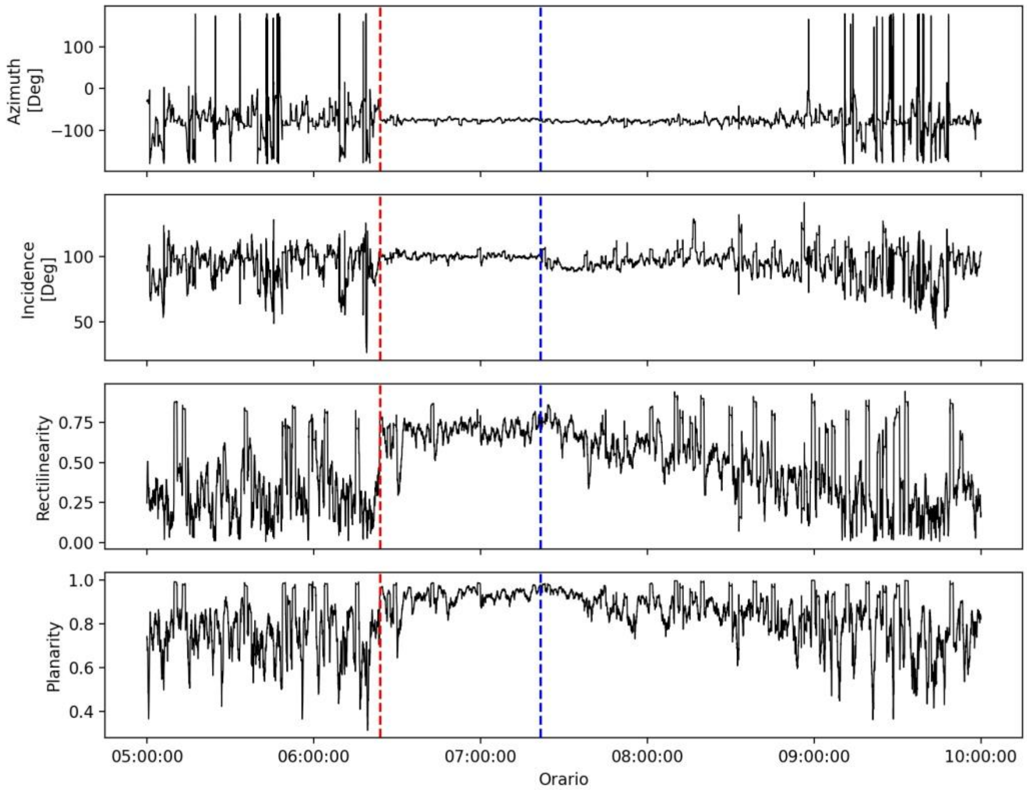

Figure S2 Polarization parameters in the 0.2-0.5 Hz frequency band for the October 9, 2022 event. From top to bottom: polarization azimuth; angle of incidence; rectilinearity and planarity.

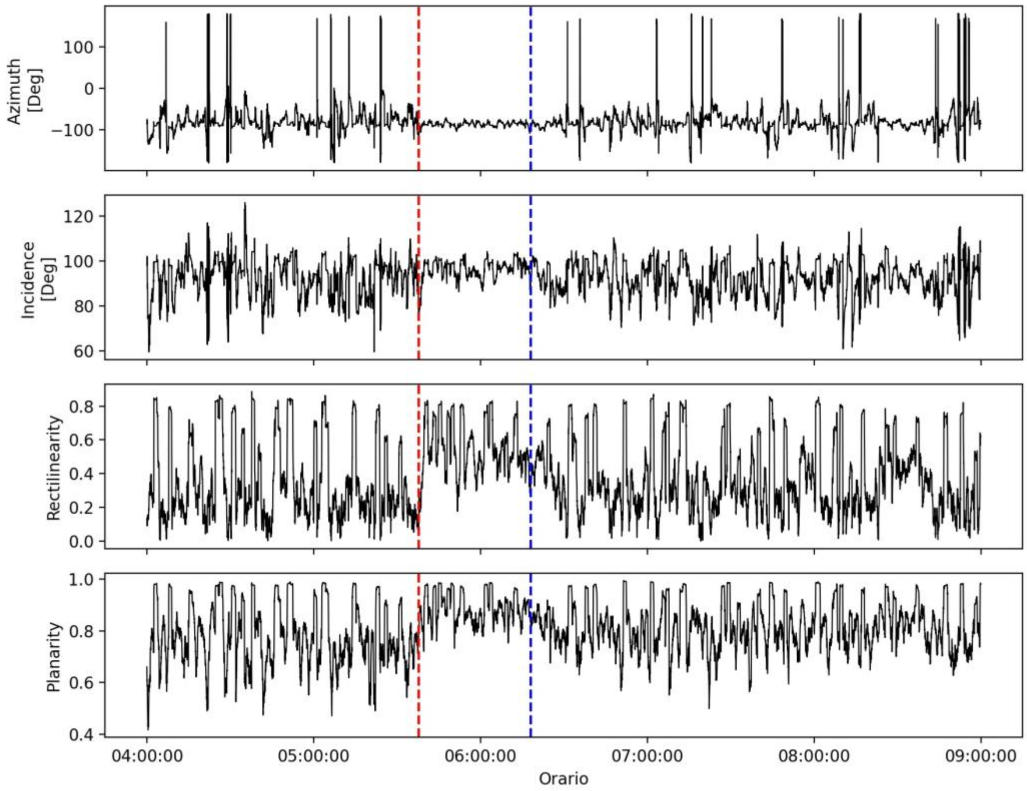

Figure S3 Polarization parameters in the 0.2-0.5 Hz frequency band for the November 16, 2022 event. From top to bottom: polarization azimuth; angle of incidence; rectilinearity and planarity.

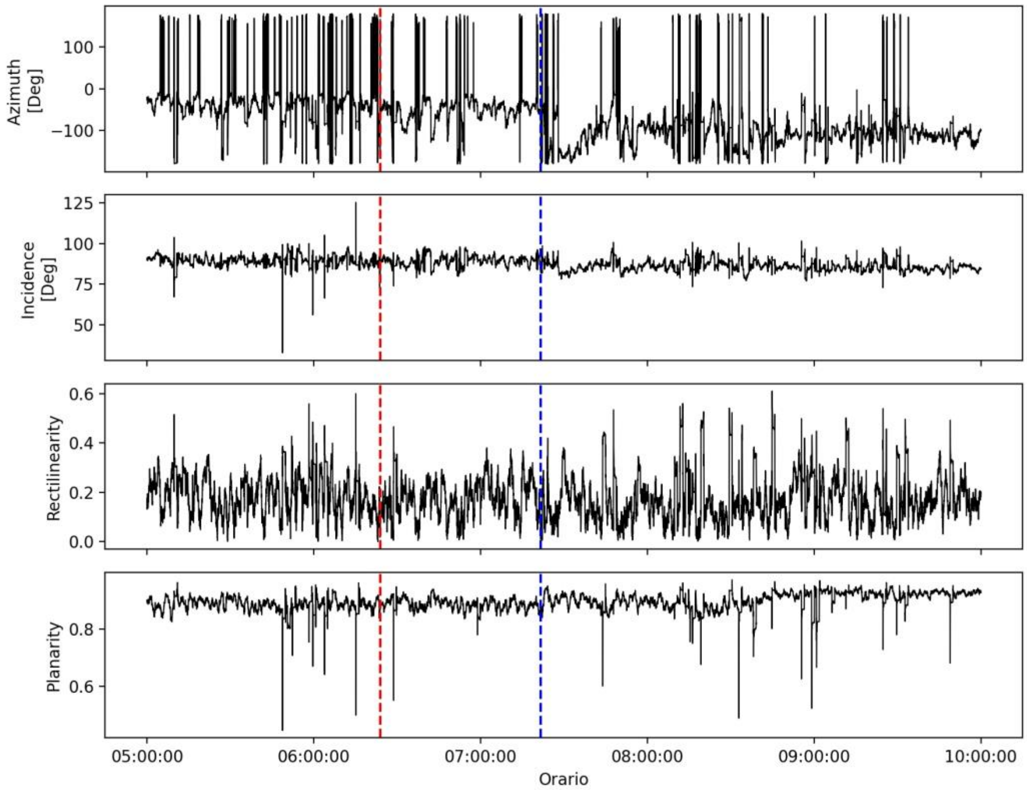

Figure S4 Polarization parameters in the 1.4-2.3 Hz frequency band for the October 9, 2022 event. From top to bottom: polarization azimuth; angle of incidence; rectilinearity and planarity.

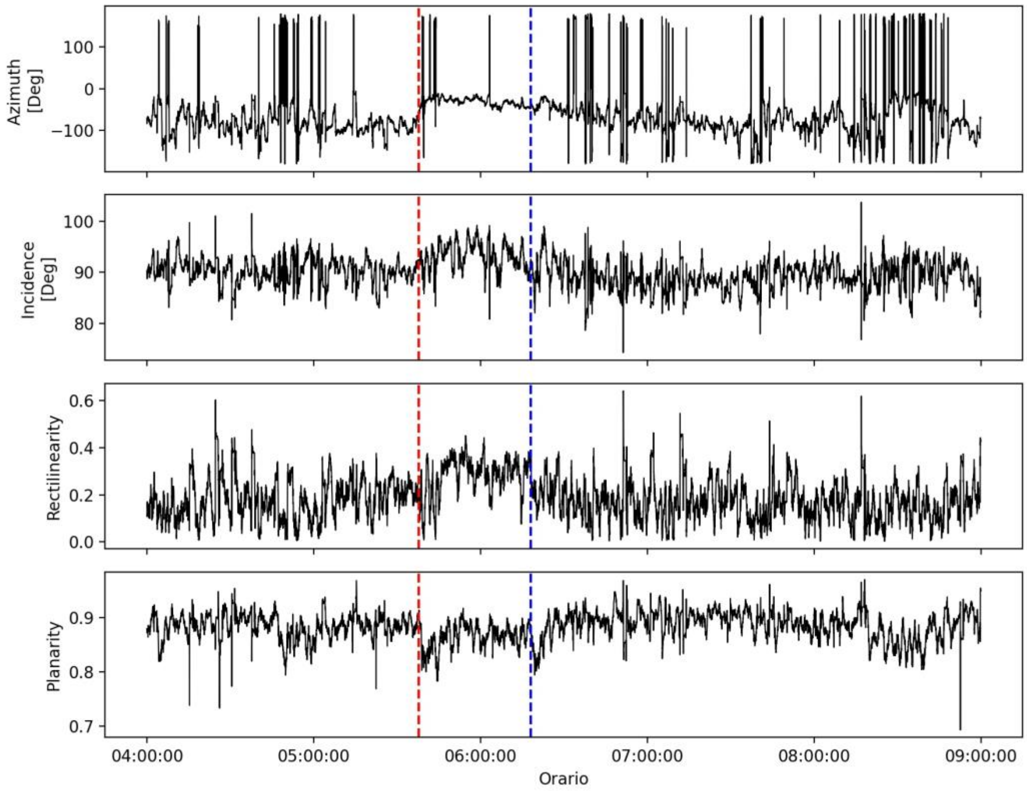

Figure S5 Polarization parameters in the 1.4-2.3 frequency band for the November 16, 2022 event. From top to bottom: polarization azimuth; angle of incidence; rectilinearity and planarity.

Correlation between seismic and thermal precursors

We calculated the correlation coefficients between the RSAM in 1-7 Hz frequency band and T-Idx for the two overflow case studies analyzed in this study. To remove the peaks caused by explosions, we replaced the values within the segment containing the explosion signal with the last value observed before the start of the explosion signal, in both time series. Subsequently, we normalized the time series (Fig. S6 and Fig. S7). Finally, using the NumPy library's function, we computed the Pearson product-moment correlation coefficients between the two normalized time series. For the event that occurred on October 9, 2022, we selected a specific time window from 6:00 to 7:00 UTC, which encompassed the onset of the seismic and thermal precursors. The obtained correlation coefficient was 0.88 (Fig. S6).

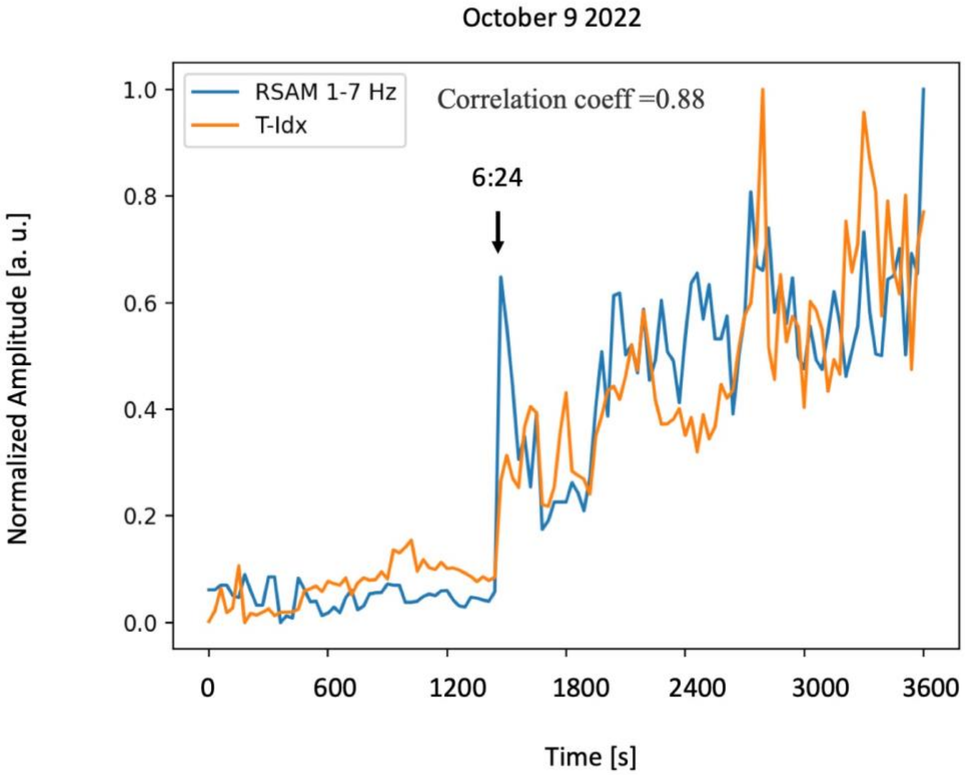

Figure 6 The Pearson product-moment correlation coefficients between the RSAM in 1-7 Hz frequency band and T-Idx for the overflow event on October 9, 2022 calculated using NumPy library. Correlation Coefficient = 0.88. The time range is 6:00-7:00 UTC. Seismic and thermal precursors start at 6:24 am and the overflow starts at 7:22 am.

We repeated the same analysis for the November 16, 2022 event by selecting the time window between 5:00 and 6:00 UTC (Fig. S7). For this event we obtained a correlation coefficient = 0.93.

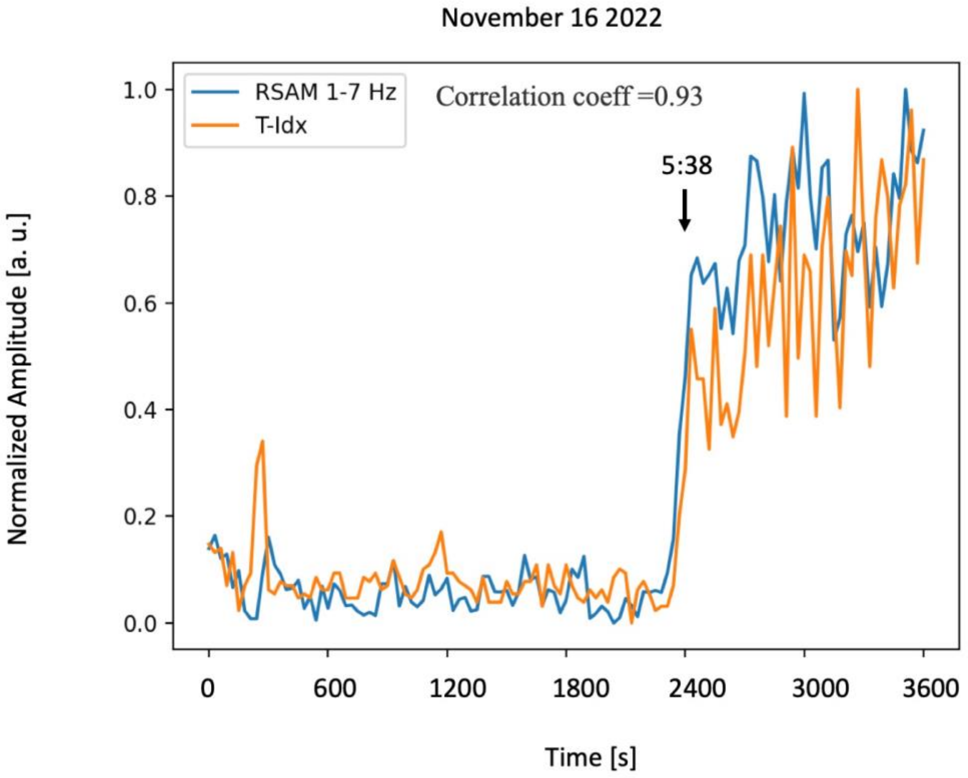

Figure 7 The Pearson product-moment correlation coefficients between the RSAM in 1-7 Hz frequency band and T-Idx for the overflow event on November 16, 2022 calculated using NumPy library. Correlation Coefficient = 0.93. The time range is 5:00-6:00 UTC. Seismic and thermal precursors start at 5:38 am and overflow starts at 6:18 am.
